# Encephalopathy in COVID-19 Patients

**DOI:** 10.7759/cureus.16620

**Published:** 2021-07-25

**Authors:** Parth Shah, Jinish Patel, Noha N Soror, Ritha Kartan

**Affiliations:** 1 Internal Medicine, Western Reserve Health Education/Northeast Ohio Medical University (NEOMED), Warren, USA; 2 Internal Medicine, Trumbull Regional Medical Center/American University of Antigua, Warren, USA; 3 Pulmonary and Critical Care, Trumbull Regional Medical Center/Northeast Ohio Medical University (NEOMED), Warren, USA

**Keywords:** covid 19, covid-19-related encephalopathy, cytokine storm syndrome, medical intensive care unit (micu), high dose corticosteroids

## Abstract

The clinical presentation of coronavirus disease 2019 (COVID-19) has a wide spectrum, ranging from asymptomatic patients to severe presentations with acute respiratory distress syndrome (ARDS), kidney injury, stroke, electrolyte imbalance, and multi-organ failure. Encephalopathy and encephalitis are devastating severe acute respiratory syndrome coronavirus‐2 (SARS‐CoV‐2) virus‐associated central nervous system complications.

We reported a case of a 67-year-old male who was admitted to the hospital for the management of COVID-19 pneumonia. Due to worsening hypoxia, the patient was transferred to ICU and was intubated. On examination, he was aphasic and noted to have right-sided hemiplegia with left-sided hemiparesis on day 4. CT scan of the head without contrast and MRI findings were suggestive of acute necrotizing encephalopathy secondary to intracranial cytokine storm caused by viral infection. The patient was treated with intravenous immunoglobulin (IVIG), and high dose corticosteroids, with clinical improvement in the right-sided hemiparesis on day 5. A repeat MRI brain revealed decreased edema.

The pathogenesis of encephalopathy associated with COVID-19 may be multifactorial. Postulated mechanisms include hypoxic/metabolic changes produced by the intense inflammatory response due to cytokine storm and neurotropism. Cytokine storm causes hypoxia and metabolic insults that result in global dysfunction of the brain. Altered consciousness, ranging from mild confusion, delirium, to deep coma, are some of the cardinal clinical features. The most common imaging finding on MRI T2-weighted fluid-attenuated inversion recovery (MRI T2/FLAIR) includes symmetric, multifocal lesions with invariable thalamic involvement. Other commonly involved locations include the brainstem, cerebral white matter, cortical and subcortical white matter, and cerebellum. In a few case reports, cerebrospinal fluid (CSF) analysis has shown the presence of the virus. Management of encephalopathy in COVID-19 patients involves supportive care including supplemental oxygen therapy and immune modulators. Immune modulation therapy including high-dose corticosteroids and IVIG have been shown to improve outcomes in these patients.

## Introduction

An outbreak of the coronavirus disease 2019 (COVID-19) caused by severe acute respiratory distress syndrome coronavirus 2 (SARS-CoV-2) began in the Hubei province of China resulting in a global health emergency. Due to the rapid spread of infection in the community, and lack of a clear understanding of the disease presentation and suboptimal management options resulted in increased morbidity and mortality.

The clinical presentation of COVID-19 has a wide spectrum, ranging from asymptomatic patients to severe presentations with acute respiratory distress syndrome (ARDS), kidney injury, stroke, and multi-organ failure [1]. The most common presenting features include fever, cough, rhinorrhea, sore throat, dyspnea, headache, myalgia, nausea, abdominal pain, and diarrhea [1]. A significant proportion of patients have been shown to present with various neurological findings. Literature suggests that approximately 50% of patients presenting with COVID-19 can have neurological manifestations. Some of the commonly presented neurological features include anosmia, dysgeusia, headache, myalgias, dizziness, altered mental status, and stroke [2-4]. According to a study by Mao et al., the patients with severe infection had more neurological manifestations and presented with complications such as cerebrovascular diseases, altered consciousness, and skeletal muscle injury [2]. The proportion of patients presenting with neurological complications in SARS-CoV-2 infection irrespective of the degree of severity is significantly higher.

Among the neurological complications, encephalopathy is seen in COVID-19 patients, especially in the severely ill population [3]. In a recent review of hospitalized COVID-19 patients, the incidence rate of encephalopathy was noted to be around 8.7% [5]. Encephalopathy refers to diffuse brain dysfunction, usually manifesting as altered mental status. A proportion of patients may present with additional features such as cognitive deficits, seizures, headache, asterixis, or myoclonus. The pathogenesis of encephalopathy associated with COVID-19 may be multifactorial. Postulated mechanisms include hypoxic/metabolic changes produced by the intense inflammatory response due to cytokine storm and neurotropism.

We describe a patient here who developed encephalopathy, a neurological complication secondary to COVID-19 infection. We aim to present the clinical features, pathophysiology, and management options for COVID-19 patients with encephalopathy.

## Case presentation

**Figure 1:**
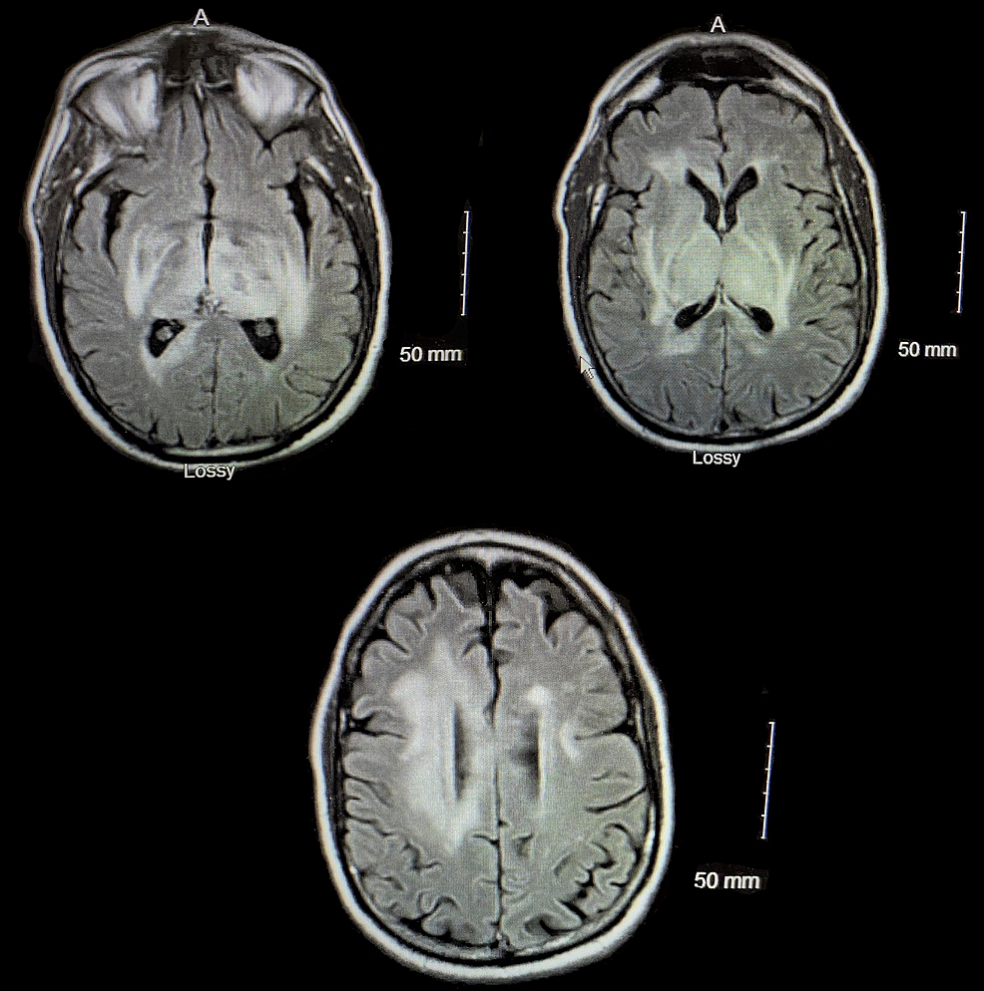
MRI of the brain showing diffuse edema and micro-hemorrhages.

A 67-year-old male with a past medical history of hypertension presented to the emergency department (ED) with new-onset dyspnea. He is a physician who was recently exposed to COVID-19 patients. CT scan of the chest revealed bilaterally developing small infiltrates and mild peripheral ground-glass opacities consistent with COVID-19. The diagnosis of COVID-19 was confirmed by reverse transcription-polymerase chain reaction (RT-PCR). He was discharged with instructions to self-quarantine at home for 14 days, as he was hemodynamically stable and saturating well on room air. Three days later, he presented with worsening dyspnea. Repeat CT chest revealed interval worsening of multilobar pneumonia with worsening consolidation bilaterally. On admission, he was hypoxic with oxygen saturation of 78% on room air which improved to 96% with 6 L of oxygen by nasal cannula. His worsening hypoxia with oxygen saturation of 82% prompted his transfer to ICU for intubation and mechanical ventilation. He was gradually improving over the next three days and then was put on pressure support trials. During the physical examination, he was aphasic and noted to have right-sided hemiplegia with left-sided hemiparesis. CT head without contrast demonstrated focal hypodensities within the right caudate as well as subtle hypodensities in the left basal ganglia, thalamus, pons, and temporal lobe. Subsequently, MRI of the brain demonstrated scattered microhemorrhages with edema in deep nuclei suggesting acute necrotizing encephalopathy related to intracranial cytokine storm secondary to viral infection (Figure 1). Electroencephalogram (EEG) demonstrated mild to moderate generalized slowing of waves. Analysis of cerebrospinal fluid (CSF) revealed elevated protein. He was switched from dexamethasone to high-dose corticosteroids. Intravenous immunoglobulin (IVIG) and levetiracetam were added to his management while sedation was discontinued. Five days after IVIG, he improved clinically with minimal right-sided hemiparesis. Repeat MRI brain revealed decreased edema. He continued to improve on the current treatment. Eventually, he underwent tracheostomy and percutaneous endoscopic gastrostomy (PEG) with tube placement. He was discharged to a long-term acute care facility for further management and rehabilitation.

## Discussion

Pathophysiology

The pathogenesis of encephalopathy associated with COVID-19 may be multifactorial. In order to maintain the normal function of neurons, optimal conditions with a balanced environment of water, electrolytes, metabolites, and other chemicals such as neurotransmitters are required [6]. Severely ill COVID-19 patients are prone to the same causes of toxic-metabolic encephalopathies as other entities. The virus is known to cause a cytokine storm syndrome characterized by excessive production of pro-inflammatory markers including tumor necrosis factor alpha (TNF alpha), interleukin-6 (IL-6), and interleukin-1beta (IL-1β) [4]. In addition to cytokine storm, it causes hypoxia and metabolic insults that result in global dysfunction of the brain [7]. There is also some evidence that the novel coronavirus has neurotropism [8]. SARS-CoV-2 uses spike proteins on the viral surface to bind to the angiotensin-converting enzyme 2 (ACE2) receptor on host cells, similar to SARS-CoV-1. Viral cellular tropism in humans is determined by the presence of ACE2 receptor on cells. In humans, ACE2 is expressed in multiple cell types including endothelium, lungs, kidney, and central nervous system (CNS) [9]. One proposed mechanism for neurotropism is direct spread across the blood-brain barrier via ACE2 on vascular endothelial cells [9]. Another plausible mechanism for SARS-CoV-2 entry to the CNS is through olfactory neurons, considering the isolated loss of sense of smell (anosmia) [9]. Butowt and von Bartheld reported that neurotropism may be caused by virus-induced inflammation or vascular/systemic routes rather than olfactory neurons given that the sudden loss of smell is followed by a rapid recovery which is less than one week [10].

Clinical features

Several clinical findings related to COVID-19 associated encephalopathy are decreased level of consciousness, delirium with altered attention, drowsiness, agitation, hemiplegia, hypertonia, hyperreflexia, extensor plantar response, alogia, and abulia [2-3, 11]. Nonspecific symptoms such as myalgias, headache, dizziness, anosmia, and dysgeusia were reported early in the disease process and were reported with less severe cases [4]. Although SARS-CoV-2 is associated with a wide range of neurological clinical presentations, there is not enough evidence to list a complete range of neurological manifestations.

Imaging findings

The most common imaging finding on MRI T2/FLAIR includes symmetric, multifocal lesions with invariable thalamic involvement. Other locations commonly involved include the brainstem, cerebral white matter, cerebellum, cortical and subcortical white matter [11]. T2/FLAIR hyperintensity in the periventricular area and several microhemorrhages were seen in several images [11].

Electroencephalography

The most common findings on EEG for patients with encephalopathy are generalized symmetrical slowing, and the presence of focal disturbance, which may be suggestive of COVID-19 associated encephalopathy [12]. Although, in one retrospective study of 22 patients with encephalopathy, EEG showed near-normal patterns [13].

Cerebrospinal fluid findings

Cerebrospinal fluid findings may not be specific for encephalopathy in COVID-19 patients. In a few studies, the CSF analysis revealed normal white cell count, glucose levels, and was negative for RT-PCR for the novel coronavirus [3]. In one isolated case, CSF analysis showed markedly increased levels of protein (>200 mg/dL) and pro-inflammatory cytokines, IL-6, IL-8, IL-10, interferon-gamma-induced protein 10 (IP-10), and TNF-alpha, however, real-time RT-PCR was negative [14]. Garg et al. reported the first case of meningitis/encephalitis associated with SARS-CoV-2 in which RT-PCR of CSF was positive even though RT-PCR of the nasopharyngeal swab was negative [11]. 

Management

Management of encephalopathy in COVID-19 patients involves supportive care including supplemental oxygen therapy and immune modulators [15]. Immune modulation therapy including high-dose corticosteroids (IV methylprednisolone 500 mg-1 g/day for five days) and IVIG ( 0.1-0.5 g/kg/ day for 5-15 days) as opposed to antiviral therapy is required in systemic inflammatory response caused by SARS-CoV-2 [16-17]. Repeated plasmapheresis has also been shown to improve consciousness and decrease pro-inflammatory marker levels in the serum [18]. Often encephalopathic patients are admitted to the ICU for management, generally requiring mechanical ventilation. Anti-epileptic medications should be started as abortive and prophylactic therapy in critically ill patients with altered mentation, convulsions, or subtle twitching. However, adverse effects and drug interactions should be monitored as antiepileptic medications can have significant respiratory/cardiac adverse effects [19].

## Conclusions

COVID-19 patients with comorbid neurological disease, including stroke, have significantly higher rates of mortality, delirium, and disability. Some patients with delirium and/or neurological symptoms lead to prolonged sedation and mechanical ventilation resulting in worsening of the prognosis. Plasmapheresis and corticosteroids have shown improvement in consciousness and disease progression.
